# Age-related differences in affective responses to and memory for emotions conveyed by music: a cross-sectional study

**DOI:** 10.3389/fpsyg.2013.00711

**Published:** 2013-10-16

**Authors:** Sandrine Vieillard, Anne-Laure Gilet

**Affiliations:** ^1^Laboratoire de Psychologie (EA 3188), Psychology, Université de Franche-ComtéBesançon, France; ^2^Laboratoire de Psychologie des Pays de la Loire (EA 4638), Université Nantes Angers Le MansNantes, France

**Keywords:** aging, musical emotions, emotional responses, facial muscle activity, incidental recognition, positivity effect

## Abstract

There is mounting evidence that aging is associated with the maintenance of positive affect and the decrease of negative affect to ensure emotion regulation goals. Previous empirical studies have primarily focused on a visual or autobiographical form of emotion communication. To date, little investigation has been done on musical emotions. The few studies that have addressed aging and emotions in music were mainly interested in emotion recognition, thus leaving unexplored the question of how aging may influence emotional responses to and memory for emotions conveyed by music. In the present study, eighteen older (60–84 years) and eighteen younger (19–24 years) listeners were asked to evaluate the strength of their experienced emotion on happy, peaceful, sad, and scary musical excerpts (Vieillard et al., 2008) while facial muscle activity was recorded. Participants then performed an incidental recognition task followed by a task in which they judged to what extent they experienced happiness, peacefulness, sadness, and fear when listening to music. Compared to younger adults, older adults (a) reported a stronger emotional reactivity for happiness than other emotion categories, (b) showed an increased zygomatic activity for scary stimuli, (c) were more likely to falsely recognize happy music, and (d) showed a decrease in their responsiveness to sad and scary music. These results are in line with previous findings and extend them to emotion experience and memory recognition, corroborating the view of age-related changes in emotional responses to music in a positive direction away from negativity.

## Introduction

Research on age differences in emotion processing has been mostly restricted to visual stimuli (e.g., facial expression, video, words, and pictures) but a growing body of research converges in indicating that music also serves as a powerful emotional trigger. For example, Blood et al. (1999) have shown that classical musical stimuli which are selected to elicit intensely pleasant emotional responses engage neural networks that are implicated in reward. Neuropsychological studies have also demonstrated that the amygdala is recruited when processing scary music (e.g., Gosselin et al., 2005, 2007). Physiological data have also put in evidence that music is a strong emotion inducer (e.g., Khalfa et al., 2002). Furthermore, music has the clear advantage of maintaining attention toward the emotions conveyed because it does not allow perceptual attention to be redirected (except if the listener takes off his/her headphones). For these reasons, music appears as a viable method to test age-related changes in emotion processing.

Among the past studies that have addressed the question of age-related changes in musical emotion processing, most of them have focused on the people's ability to *recognize* musical emotions (Allen and Brosgole, 1993; Laukka and Juslin, 2007; Drapeau et al., 2009; van Tricht et al., 2010; Lima and Castro, 2011; Vieillard et al., 2012). For instance, Drapeau et al. (2009) compared healthy elderly adults and elderly adults with Alzheimer's disease in their ability to rate the extent to which the selected musical stimuli communicating happiness, peacefulness, sadness and fear (Vieillard et al., 2008) expressed each of these four emotions. Their findings showed that recognition performances of healthy older adults were relatively preserved with the highest recognition accuracy for happy stimuli. Laukka and Juslin (2007) compared young and older adults' ability to recognize anger, fear, happiness, sadness, and neutrality in short melodies performed on an electric guitar with different degrees of expressivity. In their study, the participants judged the emotional expression of each stimulus in a forced choice task comprised of anger, fear, happiness, sadness, neutral, and “other emotion” alternatives. Compared to young adults, older adults were less accurate in recognizing negative emotions such as sadness and fear, but their ability to recognize other emotion categories was spared. More recently, Lima and Castro (2011) went one step further by examining age-related changes in emotion recognition among three age groups of participants (i.e., young, middle-aged, and old). Selecting the same musical excerpts as those used in Drapeau et al. (2009)'s study, the authors asked the participants to judge the emotional intensity *perceived* for each stimulus on four rating scales (i.e., happiness, sadness, peacefulness, and fear) presented simultaneously. Consistent with previous findings, the authors found an emotion-specific age-related change characterized by a stable recognition of happiness and peacefulness categories but a gradual decline in responsiveness to sad and scary music from young adulthood to older age.

As an alternative to the hypotheses of age-related differences in cognitive functioning^1^ or hearing loss^2^, the above findings have been interpreted as being the result of a combination of age-related changes in brain structure and functioning and in motivational goals. On the one hand, empirical evidence suggest that the observed decline found in older adults in negative emotion recognition may be the result of a linear reduction in the volume of the amygdala (e.g., Zimmerman et al., 2006) and/or of a decrease of the reactivity to negative information in the amygdala (e.g., Mather et al., 2004). On the other hand, Socioemotional Selectivity Theory (Carstensen et al., 1999; Carstensen, 2006) suggests that the decline in the recognition of negative emotions would reflect a motivational shift toward emotionally meaningful goals due to an increased awareness of the limited perspective of time. This so-called “positivity effect” which refers to all combinations of *enhanced* processing of positive information and *reduced* processing of negative information, has been thought of as an emotion regulation strategy to preserve high levels of well-being in later life. In a recent literature review, Reed and Carstensen (2012) showed that this positivity effect (1) requires cognitive resources (e.g., Mather and Knight, 2005), (2) is sensitive to the experimental context (Kensinger et al., 2002; Grühn et al., 2005), and (3) is adaptive, i.e., it emerges when emotional well-being is prioritized. Consequently, the authors claimed that the positivity effect would represent a controlled shift in attentional resources rather than an *automatic* process associated with the neuronal degeneration in brain regions. Such view is compatible with the idea that the positivity effect may have a cognitive counterpart.

Recently, Vieillard et al. (2012) conducted a study in order to test for age-related differences in the psychological structure of musical emotions, and to assess whether these changes may be associated with a decrease in emotional complexity. In this research, younger and older participants were presented with musical excerpts conveying different emotions such as happiness, peacefulness, sadness, and fear (Vieillard et al., 2008). Participants were asked to perform an emotional judgment task using different rating scales (i.e., valence, hedonic value, arousal, and liking) as well as a free categorization task in which they freely created emotional categories based on the perceived acoustical cues. Findings showed age-related differences characterized by a reduced processing of arousal for scary music, an increased focus on happy music, and an emotional dedifferentiation corresponding to a decrease in differentiation between the arousal and valence dimensions. Such results have been explained within the framework of the Dynamic Integration Theory (Labouvie-Vief, 2009; Labouvie-Vief et al., 2010) postulating that the degradation of emotional complexity would be the cognitive counterpart of the older adults' attempt to maximize positive affects and minimize negative affect in order to preserve well-being.

In short, the studies reviewed above show converging evidence for a positivity effect in how emotions in music are perceived, categorized, and recognized with advancing age. However, several questions remain open. First, little is known about age-related changes regarding the emotions *experienced* while listening to music. Previous findings have suggested that participants were more accurate in their judgment of intended emotions in musical excerpts when focusing on their own emotional experience (Vieillard et al., 2008). One of the goals of the present study was thus to investigate the influence of aging on emotion processing while being personally engaged in musical listening. This is an important question because it has been suggested that emphasizing on emotion rather than on knowledge may be more meaningful to older adults (e.g., Mikels et al., 2010). Second, as far as we know, the possibility of age-related changes in memory recognition for positive musical stimuli has yet to be examined. Past research showed that age-related changes in emotional goals influence memory. A positivity effect in memory recognition tasks has already been shown in older adults for affective pictures (e.g., Charles et al., 2003; Mather and Carstensen, 2005) and for words (e.g., Kensinger, 2008). The main explanation was that memory can work as an elaborative process to regulate emotions such that the older adults' goal to maintain well-being would influence mental constructions of the past and thus lead to a positivity effect in the way they remember events. In line with this hypothesis, it has been showed that memory for negative pictures decreased in older adults both in recall and recognition tasks (e.g., Charles et al., 2003). Kensinger (2008) found a positivity effect in older adults for non-arousing words, explaining this as an age-related difference in the way positive information was primarily processed as a function of differences in motivational goals at each age level. To date, the question remains open whether age-related differences in the elaborative processing of memory may be observed in a non-verbal channel of emotion communication such as music. Third, to our knowledge, no previous study has investigated age differences in facial muscle activity when listening to music. Past studies examining age-related differences in facial expressiveness have found that young and older adults express similar patterns of facial responding to visual stimuli such as emotional scenes, objects or faces (Reminger et al., 2000; Smith et al., 2005; Bailey et al., 2009). However, older adults compared to younger adults may exhibit diminished reactivity in facial expressiveness (Smith et al., 2005; Burriss et al., 2007). The reduction of facial expressiveness in the elderly has been thought to be a possible consequence of general physiological losses in the nervous system. However, another explanation suggests that the reduction of facial expressiveness may reflect an attempt to regulate emotion since facial expression may be motivationally driven (Smith et al., 2005). Given such emotion regulation hypothesis and in line with the embodiment theory of emotion (Niedenthal et al., 2005), one can imagine that facial expressions may not only help to down-regulate emotion (by displaying less facial expressions), but also allow to modify the emotional reaction (by displaying a facial expression contrary to what one feels). In this perspective, facial electromyogram (EMG) appears as an interesting indicator of whether there is congruence between facial expressivity and musical emotion in both young and older adults, or do older adults express positive facial expression as a means to counteract negative emotions.

There is an agreement to consider that emotional responding is a multi-component process, giving rise to affective experiences, physiological adjustments and expressive behaviors (e.g., Scherer, 2005). These various aspects of emotion may be differentially influenced by age. Therefore, a unifying view of these changes is necessary to give more insight into the lifespan developmental course of emotion, particularly in the musical domain in which this topic has remained unexplored. To this end, we focused on different indexes of emotional response to music, namely subjective experience, emotion expression and memory recognition for musical excerpts that conveyed different emotions.

Finally, although the positivity effect has been observed across a number of experimental paradigms such as dot-probe tasks (Charles et al., 2003), eye-tracking paradigms (Isaacowitz et al., 2006a,b), working memory (Mikels et al., 2005), memory recognition and free recall tasks (Charles et al., 2003), and across a variety of stimuli (e.g., pictures, word lists, facial expressions), the robustness of this phenomenon has been mainly demonstrated through the visual channel of emotion communication. Consequently, research regarding the effect of age on emotion processing in music is needed to test for the generalizability of the positivity effect.

### Current research

The present study was designed to further extend previous studies and expand experimental designs to the domain of music. Our aim was twofold: first, to investigate the effects of aging on the emotion felt when listening to music and second, to address age-related differences on memory recognition for musical excerpts as a function of their intended emotion. To this end, we used a set of musical stimuli expressing happiness, peacefulness, sadness, and fear which were all controlled for valence and arousal (Vieillard et al., 2008). We used a rating task focused on the emotion experienced by the participants rather than on the emotion recognized by the participants. In order to address more extensively the effect of aging on the experienced emotion, we designed the experiment so that the subjective report of the intensity of the emotion felt was coupled with a recording of participants' facial expressions. These particular indexes were chosen since past research has shown that facial expressions, measured by the corrugator (i.e., frowning) and zygomatic (i.e., smiling) muscle activity, were mostly related to the valence in music: positive emotions generally lead to increased zygomatic activity, while negative ones were associated more with increased corrugator activity (e.g., Witvliet and Vrana, 2007; Khalfa et al., 2008). Because it has been suggested that facial EMG may also be voluntarily modulated to serve emotion regulation goal (Smith et al., 2005), we used this index to examine to what extent and how older adults show positive or negative facial expression as a function of musical emotions.

Based on the hypothesis postulating a motivated attention toward positivity with advancing age, and given the view that situations relevant to a person's motivational goals may elicit more intense emotional experience (e.g., Charles and Piazza, 2007), we expected that older adults, compared to younger adults, would judge their feeling as more intense when listening to positive musical excerpts (especially happy music that is more arousing than peaceful one) than when listening to negative musical excerpts. Since no age-related changes in facial expressivity were found in previous research (e.g., Levenson et al., 1991; Tsai et al., 2000; Magai et al., 2006), we also expected older adults to be spared in their facial expressions (i.e., corrugators and zygomatic muscle activity). More specifically, if older adults have spontaneous facial activity, it is expected that they would display a greater zygomatic activity for positive music in comparison with their younger counterparts. At the same time, it is also expected that older adults would display voluntary facial expression as a tool to manage emotion. In this hypothesis, older adults would show reduced expressivity or incongruent expression, in particular in response to negativity. Moreover, in view of the scarce data available on the influence of aging on memory recognition for musical excerpts that convey different emotions, and because the memory elaborative processes for music are based on more abstract information than those involved in the memory for visual and autobiographical material, it is difficult to predict the nature of the effects likely to be observed. Consequently, we conducted an exploratory approach to test whether the positivity effect may be generalized to memory recognition for musical excerpts conveying different emotions. We expected that compared to younger adults, older adults should better recognize positive musical excerpts than negative ones.

## Method

### Participants

A total of 40 native French speaking volunteers (22% amateur musicians^3^) participated in the present study. Exclusion criteria included the presence of uncorrected hearing, medical or psychiatric antecedents, psychotic symptoms, and history of substance abuse. As a result, the data of 18 young adults (19–24 years, *M* = 21 years; 61 % females) and 18 older adults (60–84 years, *M* = 66 years; 83% females) was analyzed. Younger and older adults were recruited respectively at the psychology department of the University of Franche-Comté and through senior social programs in Besançon. Participants did not receive financial compensation for their participation.

### Apparatus

Participants were tested individually in a quiet room at stable ambient temperature at the University. Facial muscle activity was monitored continuously during the listening and rating phases using an MP150 Biopac system (Biopac Systems, Inc., Goleta, CA) at a sampling rate of 500 Hz and processed using AcqKnowledge software. Eprime software (Schneider et al., 2002) was used for excerpts presentation and ratings recording. Musical excerpts were presented binaurally through Professional 240 Sennheiser headphones.

### Materials

Forty short musical excerpts, computer-generated in a piano timbre and taken from Vieillard et al. (2008) set of unfamiliar musical stimuli were selected for their power to convey four distinct emotions (i.e., happiness, peacefulness, sadness, and fear). Musical excerpts were controlled for their valence (unpleasant vs. pleasant) and arousal (low vs. high). Each emotion category included ten musical excerpts that lasted an average of 10 s. The happy excerpts were written in a major mode at an average tempo of 137 Metronome Markings (MM range: 92–196), with the melodic line lying in the medium high pitch range (the pedal was not used). The peaceful excerpts were composed in a major mode, had an intermediate tempo (mean: 74 MM, range: 54–100), and were played with pedal and arpeggio accompaniment. The sad excerpts were written in a minor mode at an average slow tempo of 46 MM (range: 40–60), with the pedal. The scary excerpts were composed with minor chords on the third and sixth degree, hence implying the use of many out-of-key notes. Although most scary excerpts were regular and consonant, a few had irregular rhythms and were dissonant. Their tempo varied from 44 to 172 MM. Examples can be heard at www.brams.umontreal.ca/peretz. A previous study that was conducted to examine the effect of age on emotion perception in music demonstrated that older listeners successfully distinguished happiness, peacefulness, sadness, and fear conveyed by these musical excerpts (Vieillard et al., 2012). In a study phase described below, participants were presented with 20 musical excerpts (i.e., 5 happy, 5 peaceful, 5 sad, and 5 scary) and were instructed to indicate what they experienced in terms of Emotional Intensity. The 20 remaining musical excerpts were then used as lures in the incidental recognition task.

### Procedure

The experiment was divided into two sessions separated by an interval of ~1 week. During the first session, participants completed a consent form and were asked about their age, musical listening, education level, self-reported health, visual and auditory acuity, and medical history. Auditory perception was controlled using free AudioTest software (www.cotral.com). More specifically, it was assessed by presenting pure tones at intervals between 500 and 8000 Hz to both ears through Professional 240 Sennheiser headphones. For each participant, the lowest sound pressure level at which each frequency was detected was recorded. In addition, several tests assessing general cognitive function (MMSE, Petit et al., 1998), fluid intelligence (Raven's progressive matrices, set I, Raven et al., 1998, updated 2003), and working memory (letter-number sequencing from WAIS-III, Wechsler, 2000) were administered. The first session lasted about an hour.

During the second session, physiological sensors were attached while the participants sat comfortably in a quiet room in the presence of the experimenter. To prevent participants from focusing on their facial muscles, they were informed that the electrodes placed on their face were used to record their electrodermal activity during the experiment. At the beginning of the session, two musical excerpts different from those used in the experiment were used in order to adjust the volume of the headphones for each participant. In the study phase, two practice excerpts (1 happy and 1 peaceful) following by 20 excerpts (5 of each emotion) were then presented binaurally. After each trial, participants were asked to rate the intensity of the emotion felt using a 10-point scale ranging from 0 “weak” to 9 “strong.” Facial muscle activity was also recorded. The excerpts were presented in two pseudo-randomized orders that were created to ensure that no more than two excerpts of the same emotion category were presented consecutively. Each musical excerpt was preceded and followed by two baseline periods of at least 10 s of silence.

Before the incidental recognition task, participants completed two questionnaires to assess depression (BDI-II; Beck et al., 1998) and anxiety (STAI; Spielberger, 1993). In the recognition task participants were asked to indicate whether an excerpt had been heard before (“old”) or not (“new”) by pressing the appropriate key. In this phase, 40 musical excerpts (i.e., 20 old excerpts and 20 new excerpts) were randomly presented.

Finally, participants were instructed to listen to the same set of 40 musical excerpts presented binaurally in a randomized order. After each musical excerpt, participants were asked to judge to what extent they experienced “happiness,” “peacefulness,” “sadness,” or “fear” using a 10-point scale ranging from 0 “not at all” to 9 “a lot.” Accordingly, each excerpt was presented four times; each presentation was associated with one of the four emotion scales. The presentation order of each musical excerpt and each rating scale was fully randomized across participants. The second session lasted about 2 h. At the end of the session, participants were fully debriefed.

### Data acquisition and transformation

Facial EMG activity (μVolts) was recorded over the left corrugator and zygomatic sites, using two pairs of 8 mm Ag/AgCl shielded electrodes filled with isotonic gel. The EMG data were band-pass filtered from 100 to 500 Hz and processed with a root mean square algorithm over 20 samples (with a 100-ms window). Recording artifacts were visually identified and discarded from the sample. These corresponded to less than 0.5% of all measurements.

## Results

### Sample characteristics

Younger adults reported more years of education than the older adults, *t*_(34)_ = −2.33, *p* < 0.05. A chi-square goodness-of-fit test (χ^2^) indicated no significant differences between age groups in the proportion of formal musical training of at least 3 years χ^2^ (1, N = 36) = 4.5, *p* > 0.05. Age groups did not differ regarding their depression, state anxiety, or trait anxiety scores^4^. Non-parametric Mann-Whitney *U*-test^5^ performed on the auditory thresholds (dB) did not show statistically significant differences between younger and older adults (*U* = 10.50, *z* = 0.77, *p* = 0.44 for 500 Hz; *U* = 0, *z* = 0, *p* = 1 for 1000 Hz; *U* = 10, *z* = 0.84, *p* = 0.40 for 2000 Hz; *U* = 8, *z* = 1.12, *p* = 0.26 for 4000 Hz; *U* = 33, *z* = 0.23, *p* = 0.81, for 8000 Hz). As was expected, younger adults scored better than older adults on fluid intelligence (Raven's progressive matrices, set I, Raven et al., 1998, updated 2003), *t*_(34)_ = −2.84, *p* < 0.05. Younger adults tended to perform better than older adults on a working memory test (Digit Span from WAIS-III, Wechsler, 2000), *t*_(34)_ = −1.94, *p* = 0.06. There were no significant age differences on self-reported health, *t*_(34)_ = −0.81, *p* = 0.42. Finally, the Mini Mental State Examination (MMSE; Petit et al., 1998) scores for the older adults suggested no apparent signs of dementia (*M* = 29.7, 28–30). Sample characteristics are detailed in Table 1.

**Table 1:** **Sample characteristics**.

	**Age group**	
	**Young (18)**	**Older (18)**
**DEMOGRAPHIC CHARACTERISTICS**		
Age (year)	19–24	61–74
Sex	61% Women	83% Women
Education (year)^*^	15 (3.17)	13 (1.57)
Musical training (proportion)	0.22	0
Self reported health (Max. 5)	4.44 (0.86)	4.22 (0.79)
**COGNITIVE SCORES**		
Advanced progessive matrices (Set 1, Max. 12)^*^	10.00 (1.91)	7.78 (2.71)
Letter-number sequencing (Max. 21)	11.89 (3.05)	10.17 (2.23)
MMSE (Max. 30)	–	29.7
**AFFECTIVE SCORES**		
Depression (BDI-II, Max 63)	8.33 (6.79)	8.67 (5.30)
State Anxiety (STAI-Y-A, Max 80)	30.55 (6.35)	27.83 (6.72)
Trait Anxiety (STAI-Y-B, Max 80)	40.83 (10.26)	40.17 (9.57)

Standard deviations are listed in parentheses. ^*^Significant difference at p < 0.05.

### Emotion intensity felt

A mixed model analysis of variance was conducted on the mean score of the Emotional Intensity Felt with Age Group (younger adults, older adults) as the between-subjects factor and Intended Emotions (happiness, peacefulness, sadness, fear) as the within-subjects factor.

**Figure 1:**
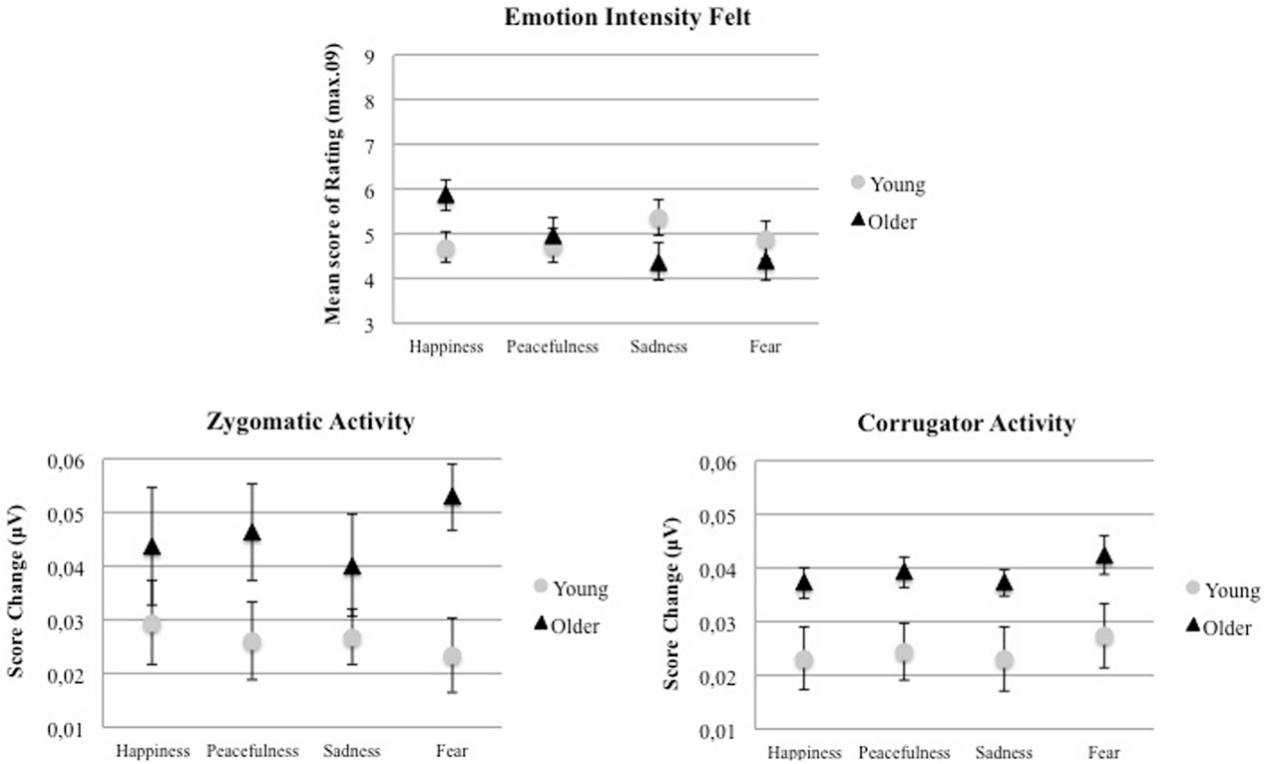
**Mean score and standard error of emotion intensity felt, zygomatic and corrugator muscle activity as a function of intended emotions (happiness, peacefulness, sadness, fear) and age group (younger adults, older adults)**.

As illustrated in Figure 1, we found a significant Age Group by Intended Emotion interaction, *F*_(3, 102)_ = 3.75, *p* < 0.05, η^2^_*G*_ = 0.06^6^. In order to test our hypothesis, we computed a planned comparison between the emotion intensity felt by young adults and that felt by older adults when listening to happy music. As expected, older adults compared to young adults reported experiencing higher emotional intensity when listening to happy music, *F*_(1, 34)_ = 5.57, *p* < 0.05, η^2^_*G*_ = 0.14. This older adults' reactivity for positivity was also confirmed by another set of planned comparisons indicating that older adults reported experiencing higher emotional activation when listening to happy music than when listening to sad music, *F*_(1, 34)_ = 7.34, *p* < 0.05, η^2^_*G*_ = 0.18, or scary music, *F*_(1, 34)_ = 8.76, *p* < 0.05, η^2^_*G*_ = 0.20, while younger adults did not. No other significant effect was found. A separate analysis with years of education, fluid intelligence scores, and working memory performances (i.e., factors that were found to be different between the two age groups) as covariates indicated that the Age Group by Intended Emotion interaction remained significant, *F*_(3, 93)_ = 3.14, *p* < 0.05, η^2^_*G*_ = 0.05.

### Facial muscle activity

Facial EMG responses were calculated as the difference between the signal (Area under the curve, μV^*^sec) over the time course of the musical excerpt and a baseline EMG level measured from 1s prior to the onset of the excerpt (time -1 to 0 s) to the beginning of the excerpt. The area under the curve was extracted within these two time windows and was averaged for each condition and for each participant. Two participants were excluded (1 younger and 1 older adults) from the initial sample due to technical problems. Analyses were then conducted on seventeen younger and seventeen older adults. The Shapiro-Wilk normality test reached significance for the sample set of EMG data meaning that the assumption of normality has to be rejected. As a result, statistical analyses were performed using non-parametric tests. First, the differences between age groups were tested separately for zygomatic and corrugator using the Mann-Whitney *U*-test. Results showed a significant effect of Age Group both for zygomatic (*U* = 42, *z* = 3.51, *p* < 0.001) and for corrugator muscle (*U* = 83, *z* = 2.10, *p* < 0.05) indicating that facial activity was more important in older adults than in their younger counterparts. Friedman repeated measures analyses of variance (RM-ANOVA) were conducted separately on zygomatic and on corrugator activity to test the effect of Intended Emotion factor for each Age Group. For zygomatic muscle, data revealed a significant effect of Intended Emotion in older adults, χ^2^ = 12.46, *df* = 3, *p* < 0.05, but not in young adults, χ^2^ = 2.01, *df* = 3, *p* = 0.57. As shown in Figure 1, older adults showed an increased zygomatic activity, in particular for scary music. Regarding corrugator activity, no significant effect of Intended Emotion was found either in older adults, χ^2^ = 3.00, *df* = 3, *p* = 0.39, or in younger adults, χ^2^ = 5.68, df = 3, *p* = 0.13.

### Incidental recognition task

Proportions of hits, false alarms, and corrected recognition scores (hits minus false alarms) are reported in Table 2. An analysis of variance was conducted on corrected recognition scores with Age Group (younger adults, older adults) as the between-subjects factor and Intended Emotion (happiness, peacefulness, sadness, fear) as the within-subjects factor. Analysis showed a significant main effect of Age Group indicating that younger adults recognized more musical stimuli than older adults, *F*_(1, 34)_ = 30.87, *p* < 0.001, η^2^_*G*_ = 0.50. There was also a significant main effect of Intended Emotion, *F*_(3, 102)_ = 4.86, *p* < 0.05, η^2^_*G*_ = 0.12, indicating that scary music stimuli were better recognized than peaceful and sad music stimuli. This was confirmed by *post-hoc* Bonferroni comparisons (*p*s < 0.05). No significant Age Group by Intended Emotion interaction was observed, *F*_(3, 102)_ = 0.27, *p* = 0.84, η^2^_*G*_ = 0.01. Younger adults' recognition performances were at chance level or above only for the highly arousing musical stimuli such as happy (52%) and scary (61%) ones, while older adults' recognition performances varied between 10 and 39% through the four intended emotions.

**Table 2:** **Hit rates, False Alarms Rates (FA) and Corrected Recognition (CR) by Emotions Catagory and Group Age**.

**Age group**	**Intended emotions**											
	**Happiness**			**Peacefulness**			**Sadness**			**Fear**		
	Hits	FA	CR	Hits	FA	CR	Hits	FA	CR	Hits	FA	CR
**YOUNG (*n* = 18**)												
*M*	0.64	0.12	0.52	0.70	0.31	0.39	0.70	0.32	0.38	0.64	0.03	0.61
*SE*	0.05	0.06	0.05	0.06	0.07	0.07	0.07	0.07	0.09	0.07	0.03	0.07
**OLDER (*n* = 18**)												
*M*	0.74	0.56	0.18	0.66	0.56	0.10	0.63	0.50	0.13	0.46	0.07	0.39
*SE*	0.05	0.06	0.05	0.06	0.07	0.07	0.07	0.07	0.09	0.07	0.03	0.07

Additional separate analyses conducted on hits and false alarms revealed that older adults generated more false alarms than younger adults, *F*_(1, 34)_ = 18.47, *p* < 0.001, η^2^_*G*_ = 0.35. There was also a significant main effect of Intended Emotion, *F*_(3, 102)_ = 22.02, *p* < 0.001, η^2^_*G*_ = 0.36, as well as a significant Intended Emotion by Age Group interaction for false alarm rates, *F*_(3, 102)_ = 4.85, *p* < 0.05, η^2^_*G*_ = 0.08. *Post-hoc* Bonferroni comparisons showed that the older listeners, compared with their younger counterparts, had more difficulty to correctly reject new happy musical stimuli (*p* < 0.05). Moreover, older adults were better to correctly reject new scary musical stimuli in comparison to all other intended emotions (*ps* < 0.001) while younger adults were better to correctly reject new scary musical stimuli in comparison to only low arousing stimuli like peaceful (*p* < 0.05) and sad (*p* < 0.05) ones. No significant effect or interaction was found for hits rates. Again, the analysis of covariance with years of education, fluid intelligence scores, and working memory performances as covariates showed that the Intended Emotion by Age Group interaction remained significant, *F*_(3, 93)_ = 3.70, *p* < 0.05, η^2^_*G*_ = 0.07.

D-prime was calculated using tables for d-prime and beta available in Hochhaus (1972) and analyzed using another mixed ANOVA. This indicates the ability to discriminate between true targets and false targets (Green and Swets, 1966), with Age Group (younger adults, older adults) as the between-subjects factor and Intended Emotion (happiness, peacefulness, sadness, fear) as the within-subjects factors. We obtained a significant main effect of Age, *F*_(1, 34)_ = 26.41, *p* < 0.001, η^2^_*G*_ = 0.44 indicating that, overall, older adults showed lower d-prime score (*M* = 0.43, *SE* = 0.15) than younger adults (*M* = 1.50, *SE* = 0.15). This suggests a lower sensitivity in the discrimination of true musical excerpts from false musical excerpts in older adults. No other significant interaction was found with d-prime as the dependent variable. The beta value that indicates the minimum level of activation necessary for a participant to respond to a true target (Green and Swets, 1966) was also calculated (Hochhaus, 1972). No significant main effect or interaction was found with beta value as the dependent variable.

### Differentiation in emotion felt

As in previous studies (Vieillard et al., 2008), we derived the best label attributed to each musical excerpt by each participant. This was done selecting the label (i.e., happy, peaceful, sad, scary) that had received the maximal rating. When the maximal rating corresponded to the label that matched the intended emotion, a score of 1 was given. When the maximal rating did not correspond to the emotion, a score of 0 was given. When the highest rating was given for more than one label, the response was considered ambivalent and received a score of 0. For example, when an excerpt was perceived eliciting both peacefulness and sadness to the same degree (e.g., with a rating of 7), it was considered as ambivalent. Best labels scores are presented in Table 3.

**Table 3:** **Mean percentage of the label that received the maximal rating of Emotion Felt by younger and older listeners as a function of the Intended Emotions**.

	**Emotion felt**				
	**Happiness**	**Peacefulness**	**Sadness**	**Fear**	**Ambivalent**
**INTENDED EMOTION**					
**Young adults**					
Happiness	**81**	5	2	1	11
Peacefulness	13	**37**	28	1	21
Sadness	1	20	**56**	10	13
Fear	10	2	9	**72**	7
**Older adults**					
Happiness	**73**	1	1	1	24
Peacefulness	5	**37**	22	7	29
Sadness	3	24	**35**	6	32
Fear	9	4	28	**34**	25

Bold indicates the match between Emotion Felt and Intended Emotions. Ambivalent responses correspond to highest ratings given to more than one label.

A mixed model analysis of variance was conducted on the mean Best Label with Age Group (younger adults, older adults) as a between-subjects factor and Intended Emotions (happiness, peacefulness, sadness, fear) and Emotion Felt (happiness, peacefulness, sadness, fear) as within-subjects factors. Significant main effects of Age Group, *F*_(1, 34)_ = 7.43, *p* < 0.05, η^2^_*G*_ = 0.18, and Emotion Felt, *F*_(3, 102)_ = 5.87, *p* < 0.001, η^2^_*G*_ = 0.14 were found. As expected, results also indicated a significant Intended Emotion by Emotion Felt interaction, *F*_(9, 306)_ = 71.45, *p* < 0.001, η^2^_*G*_ = 0.58, as well as a significant Intended Emotion by Emotion Felt by Age Group, *F*_(9, 306)_ = 5.10, *p* < 0.001; η^2^_*G*_ = 0.09. No other significant main effect or interaction was observed. We first compared the experience emotion between younger and older adults for each intended emotion. The results indicated that older adults, compared to younger adults, reported experiencing lower levels of sadness when listening to sad music, *F*_(1, 34)_ = 5.44, *p* < 0.05, η^2^_*G*_ = 0.14, but reported higher levels of sadness when listening to scary music, *F*_(1, 34)_ = 6.35, *p* < 0.05, η^2^_*G*_ = 0.16. Moreover, when listening to scary music, older adults reported experiencing lower levels of fear, *F*_(1, 34)_ = 18.78, *p* < 0.001, η^2^_*G*_ = 0.36, than their younger counterparts. The second set of comparisons was conducted to compare the emotion experienced for each intended emotion within each age group. The results showed that older adults reported similar levels of sadness and peacefulness when listening to peaceful music, *F*_(1, 34)_ = 1.92, *p* = 0.18, η^2^_*G*_ = 0.05, and sad music, *F*_(1, 34)_ = 0.85, *p* = 0.36, η^2^_*G*_ = 0.03, as well as similar levels of sadness and fear when listening to scary music, *F*_(1, 34)_ = 0.44, *p* = 0.51, η^2^_*G*_ = 0.01. Younger adults reported similar levels of sadness and peacefulness only when listening to peaceful music, *F*_(1, 34)_ = 0.73, *p* = 0.40, η^2^_*G*_ = 0.02. Happy music was the only music that primarily elicited happiness (when compared with the level of peacefulness felt) in both young and older adults, *F*_(1, 34)_ = 117.63, *p* < 0.001, η^2^_*G*_ = 0.78, and *F*_(1, 34)_ = 105.92, *p* < 0.001, η^2^_*G*_ = 0.76, respectively. The Intended Emotion by Emotion Felt by Age Group interaction remained significant when years of education, fluid intelligence scores, and working memory performances were entered as covariates, *F*_(9, 279)_ = 2.90, *p* < 0.05, η^2^_*G*_ = 0.06.

### The relationship between age and dependent measures

In order to check for any relationships between age and the different dependent measures (i.e., emotion intensity felt, physiological responses to music, recognition accuracy, and the type of emotion felt) for each of the four intended emotions, we computed a series of correlations. Because age was significantly correlated with years of education, *r*_(34)_ = −0.41, *p* < 0.05, fluid intelligence, *r*_(34)_ = −0.48, *p* < 0.05, working memory, *r*_(34)_ = −0.35, *p* < 0.05, and each of the five measures of auditory thresholds, *r*_(34)_ = 0.56, *p* < 0.001 for 500 Hz; *r*_(34)_ = 0.54, *p* < 0.001 for 1000 Hz; *r*_(34)_ = 0.61, *p* < 0.001 for 2000 Hz; *r*_(34)_ = 0.42, *p* < 0.05 for 4000 Hz; *r*_(34)_ = 0.75, *p* < 0.001 for 8000 Hz, these variables were controlled for in partial correlations. Results indicated that the mean scores of hits, corrected recognition, and d-prime for peaceful music were negatively and significantly correlated with age, *r*_(34)_ = −0.34, *p* < 0.05 for hits, *r*_(34)_ = −0.51, *p* < 0.05 for corrected recognition, and *r*_(34)_ = −0.53, *p* < 0.05 for d-prime. Similarly, age was negatively and significantly correlated with the mean score of corrected recognition and d-prime for happy music, *r*_(34)_ = −0.69, *p* < 0.001 for corrected recognition, and *r*_(34)_ = -0.65, *p* < 0.001 for d-prime, while it was positively and significantly correlated with the mean score of false alarms, *r*_(34)_ = 0.63, *p* < 0.001. Altogether, the data indicated that the older the people are, the lower their ability to discriminate studied positive musical excerpts conveying peacefulness and happiness from unstudied ones. The mean score of Beta index for sad music was also positively and significantly correlated with age, *r*_(34)_ = 0.50, *p* < 0.05, suggesting that the older the people are, the more conservative they are to discriminate studied stimuli from unstudied musical excerpts conveying sadness. Moreover, data indicated that the older the people are, the stronger their experience of sadness while listening to peaceful music, *r*_(34)_ = 0.46, *p* < 0.05, and the weaker their experience of fear while listening to scary music, *r*_(34)_ = −0.49, *p* < 0.05. No other significant correlations were found.

### The relationship between emotion intensity felt, facial muscle activity, and recognition performances

For each age group, we investigated to what extent the emotion intensity felt during the first presentation of musical stimuli was linked to the physiological responses as well as to the subsequent cognitive performances on the incidental recognition task. The relationship between physiological reactions and recognition performances was also examined. In younger adults, results indicated that the stronger the emotion intensity felt in response to sad music, the higher the hits, *r*_(14)_ = 0.54, *p* < 0.05. In older adults, results showed that the stronger the emotion intensity felt for happy music, the higher the false alarms, *r*_(14)_ = 0.54, *p* < 0.05. No other significant correlations were found.

## Discussion

In this study, we investigated how the emotional experience as well as memory recognition for musical excerpts eliciting four different emotions (happiness, sadness, peacefulness, and fear) may change with age. To this end, younger and older listeners were asked to evaluate the intensity of the emotion felt while their facial expressions (i.e., zygomatic and corrugator muscle activity) were recorded. They were then instructed to perform an incidental recognition task followed by another task in which they had to assess for each musical excerpt to what extent they experienced each of the four emotions.

As predicted, the results showed that, when presented with happy music, older adults assessed the emotion felt as more intense than their younger counterparts. The fact that older adults rated their emotional experience as significantly more intense for happy music stimuli in comparison to sad and scary music stimuli is consistent with the literature showing that aging is associated with a relative preference for positivity over negativity. This also supports the view that emotions and motivations cannot be disentangled from each other. However, the assumption that the stronger emotional experience reported by older adults while listening to happy music would be reflected in a greater zygomatic activity was not supported. Compared to younger adults, older adults showed stronger facial expressions for both corrugator and zygomatic muscles as well as for all intended emotions. This suggests that facial expressions are not exclusively aligned with the emotional state, thus raising the question of whether the general increase of facial expressiveness in older adults would simply reflect a deeper engagement in the task. However and interestingly, the current results also showed that older adults' zygomatic activity, but not for young adults', varied as a function of the intended emotions in such a way that older adults showed an increased zygomatic activity for scary excerpts but not for happy or peaceful ones. This is in line with the idea that the reaction of smiling may serve as a defensive goal in inhibiting negative feelings for older adults. It can also be argued that zygomatic activity may reflect partial facial expression of fear, but then there we should have observed a greater concomitant activity for the corrugator muscle. However, this was not observed. Taken together, these findings are consistent with the idea that older adults' facial expressions possibly reflect an attempt to regulate emotion. Further research is needed to substantiate the role of voluntary facial expressions in the older adults' response to emotions.

Consistent with our expectations, our findings indicated that older adults correctly recognized less musical excerpts than their younger counterparts. Moreover, older adults' range of performances was quite similar to that found by Kensinger (2008) with emotional words. This suggests that modality has little impact on the strength of the memory decline with aging. The results of the present study also indicated that younger adults as well as older adults better recognized negative and arousing musical excerpts (i.e., scary music) than all other excepts while producing low false alarms rates for these scary music stimuli. This corroborates the hypothesis of an increased distinctiveness of negative stimuli (e.g., Pesta et al., 2001) and extends previous studies that showed that older adults can visually detect arousing and negative stimuli as well as their younger counterparts (e.g., Magai et al., 2006; Knight et al., 2007). Our findings also gave evidence for increased false recognition for happy music stimuli in older adults but not for young adults. We found a negative relationship between age and the ability to discriminate between true and false happy musical excerpts as well as a positive relationship between the emotion intensity felt in older adults and their rate of false alarms for happy stimuli. Taken together, these findings suggest that positive emotion elicited by happy excerpts may produce an attentional bias in older adults that can lead to confusion between studied and non-studied excerpts and thus enhance the probability of false alarms. Such increase in discrimination threshold is consistent with previous studies showing that aging was associated to a higher false response rate to positive words (Fernandes et al., 2008; Piguet et al., 2008) and corroborates the idea that the reduction of distinctiveness for positive information in older adults would be the result of their liberal bias toward positivity. However, in the present study, the positivity bias is detrimental to memory accuracy.

Another main finding of the current research is that, when presented with sad and scary musical excerpts, older adults reported experiencing lower levels of sadness and fear than their younger counterparts. Correlation analyses indicated that the older the people are, the weaker their experience of fear felt while listening to scary music. This fits nicely with previous research demonstrating age-related changes in emotion recognition (Laukka and Juslin, 2007; Lima and Castro, 2011) and emotion perception (Vieillard et al., 2012) in music, and extends these studies by showing these changes also occur when participants are focused on their own emotional experience. Interestingly, compared to Lima and Lima and Castro' (2011) recognition paradigm, the personal engagement involved in the current task seems to facilitate the older adults' ability to process negative emotions. This is consistent with previous findings demonstrating that older adults benefit more from instructions encouraging to focus on emotion than on information acquisition (Mikels et al., 2010) and corroborates the view of an age-related emphasis on emotion processing. Of course, further research is needed to compare the older adults' responsiveness to musical emotions in both contexts of recognition and of emotional experience.

Given the relatively short duration of the musical stimuli used in the present study, one may argue that this could challenge their ability to induce emotions, leading participants to rate their perceived emotions rather than their felt emotions. Although studies aiming to induce felt emotions in listeners tend to use longer excerpts than those investigating perceived emotions (Eerola and Vuoskoski, 2013), we believe that the short excerpts used in our experiment also successfully induced emotions. First of all, our results indicated that participants reported moderate intensity of the emotion felt along with significant differences in facial expressivity. Furthermore, previous findings demonstrated that musical excerpts as short as 13s may recruit neural mechanisms involved in pleasant/unpleasant emotional responses (Blood et al., 1999). In the study of Vieillard et al. (2008) which used similar 10 s musical excerpts recorded in a piano timbre, listeners better recognized some intended emotions when focusing on their emotional experience rather than when focusing on the recognition of the emotion. This suggests that asking participants to focus on felt emotions increases the degree of personal engagement in musical emotion even for short musical excerpts. Taken together, these data corroborate the hypothesis that the musical emotions were not only recognized, but indeed felt.

One limitation of this study is that we used a cross-sectional design. Historical differences in the cultural system and in musical exposure may have affected young and older adults' performances differently. The observed age differences in emotional responses to music might thus reflect a cohort effect rather than an age effect. Future research would benefit from investigating this issue more thoroughly. Nevertheless, our study suggests that emotional response to music and memory recognition for musical excerpts conveying emotions show differences with advancing age. These age-related differences are characterized by a stronger emotional reactivity for happiness, an increased zygomatic activity in response to scary stimuli, an increase in false recognition for happy musical excerpts, and a decrease in responsiveness to sad and scary music. This study extends previous findings and expands them to music, a powerful channel of emotion communication. Importantly, the findings suggest that aging may cause a decrease in negative affects and an increase in positive affects even when these affects are elicited by a more abstract source of emotion that does not refer to specific events. Finally, the current data are in line with the hypothesis that older adults could use emotional coping skills acquired over the life span in order to avoid potentially negative events and maintain positive ones (Charles et al., 2001; Labouvie-Vief et al., 2010).

### Conflict of interest statement

The authors declare that the research was conducted in the absence of any commercial or financial relationships that could be construed as a potential conflict of interest.

## Footnotes

^1^Emotion categories that were less accurately recognized by older adults were not those known to be the hardest to recognize.

^2^Age groups did not differ or only differed marginally on self-reported hearing loss.

^3^The musical training was measured as the proportion of participants who received at least 3 years of formal training and who were still practicing a musical instrument without reaching professional levels.

^4^BDI-II scores indicated that 75% (*n* = 27) of the participants scored below the cut-off (score of 11) for a minimum depression symptomatology and that 19.44% (*n* = 7) of the participants scored below the cut-off (score of 19) for at least mild depression. The remaining two participants (one young and one older adult) scored between 22 and 24, which indicates moderate depression symptomatology. STAI state anxiety scores indicated that 83.33% (*n* = 30) of the participants scored below the cut-off (score of 35) for very low state anxiety, 13.89% (*n* = 5) of them scored below the cut-off (score of 45) for low state anxiety, and that 2.78% (*n* = 1) of them scored below the cut-off (score of 55) for mild state anxiety. STAI trait anxiety scores indicated that 30.56% (*n* = 11) of the participants scored below the cut-off (score of 35) for very low state anxiety, 41.67% (*n* = 15) scored below the cut-off (score of 45) for low trait anxiety, and 19.44% (*n* = 7) scored below the cut-off (score of 55) for moderate trait anxiety. The remaining two participants (one young and one older adult) scored between 57 and 58, which correspond for a high anxiety state.

^5^Because the audio test software did not provide absolute thresholds, we used the lowest sound pressure level (dB) at which each frequency was detected as an ordinal data. For this reason, the non-parametric Mann-Whitney test (independent samples) was used. Although hearing thresholds varied as a function of age, the Mann-Whitney test did not reveal significant differences between age groups probably because of the variability observed within the groups, in particular in the older adults.

^6^We computed generalized eta squared statistics (η^2^_*G*_) with the aim to yield measures of effect size comparable across a wide variety of research designs (Bakeman, 2005), regardless of whether the factor is between or within subjects. These effect-size measures provide indices of effect that are consistent with (Cohen, 1988) guidelines indicating that η^2^_*G*_ = 0.01 corresponds to a small effect, η^2^_*G*_ = 0.09 to a medium effect, and η^2^_*G*_ = 0.25 to a large effect.
